# Enhancing thermocouple’s efficiency using an electrostatic voltage

**DOI:** 10.1038/s41598-022-17750-7

**Published:** 2022-08-08

**Authors:** Tinggang Zhang

**Affiliations:** University of Alaska Fairbanks, 306 Tanana Drive, Duckering Building, Fairbanks, AK 99775 USA

**Keywords:** Thermoelectric devices and materials, Mechanical engineering, Civil engineering

## Abstract

An electrostatic voltage is formed in the proposed thermocouple by the induced electrostatic potentials at the metallurgical junctions created by the *n*- and the *p*-type legs and their semiconductor emitters that are embedded on their exterior surfaces. The usable range of the electrostatic voltage was defined and used to enhance the output power and the efficiency of the thermocouple. An analytical formulation for and the numerical simulation of the thermocouple showed that the electrostatic voltage, as an addition to the Seebeck voltage, could enhance the output power and the efficiency up to four times those of the original thermocouple design with the same leg doping densities. Furthermore, the numerical simulation showed that for a given *n*- and a given *p*-type leg doping densities, an optimal combination of the emitter doping densities could always be found so that the output power and the efficiency of the thermocouple could be enhanced up to four times those of the thermocouple without the emitters.

## Introduction

The development of an efficient and an affordable thermoelectric power generator is of broad interests both in energy efficiency (waste heat utilization) and in electricity generation, but faces significant challenges in both material science and engineering and device technologies as indicated from the historical progress of the figure of merit, *zT*, of typical thermoelectric materials^1,2^ and the reviews of advances in device technologies^3,4^. *zT* is a dimensionless parameter that characterizes the efficiency of a thermoelectric material and is calculated by $zT = \alpha ^2\,\sigma /\kappa$, where $\alpha$ is the Seebeck coefficient, $\sigma$ the electrical conductivity, and $\kappa$ the thermal conductivity. Recent progress in the synthesizing technology of polycrystal SnSe has remarkably improved the material’s efficiency so that a *zT* as high as 3.1 was obtained at 783 K^5^. This new development in thermoelectric material lights up the expectation that an effective thermoelectric generator will soon be a reality. However, many progresses remain to be made in transforming a high efficient material into an equivalent efficient device, including material synthesizing, device packaging, system integration, and working condition consideration.

Current development in device technologies has largely relied on finding high-efficient thermoelectric materials to improve the device efficiency through “tuning” a thermocouple’s geometrical size and shape, module fill factor, functional graded thermoelectric legs, and device architecture to optimize its electrical and thermal transport properties. For example, giving the electrical resistivity ($\rho$), and the thermal conductivity ($\kappa$) of the *n*- and the *p*-type legs of a thermocouple, tuning the length (*L*) and the cross-sectional area (*A*) to satisfy the relation, ${(A_p\,L_n)/(A_n\,L_p) = \sqrt{\kappa _n\,\rho _p/(\kappa _p\,\rho _n)}}$, where the subscripts *n* and *p* are leg types, an optimal *ZT* can be realized^6^. This analytical relation was confirmed in a numerical simulation which showed even when the temperature-dependent transport properties of the materials and the electrical and thermal contact resistances were accounted for^7^. The simulation further showed that a similar output power per unit module area obtained with a greater number of longer legs could be obtained with a smaller number of shorter legs when the thermal contact resistance was neglected.

Different shapes with constant cross-sectional areas along a leg had showed little effect on the output power and the efficiency of a module^8^. However, as the cross-sectional area varies along a leg improved output power and efficiency were obtained^9,10^. Among various leg shapes investigated^11–13^, pyramidal leg was predicted to have a higher output power density^10,14^ and has showed 67% increase in output power of a laboratory-scale module in comparison to a module made of cuboid legs^15^; and a hourglass shaped leg showed more than doubled electrical potential and the maximum power output compared to the cuboid leg under the given boundary conditions in the finite element simulation^16^. These improvements are mainly attributed to the reduction of the thermal conductance of the legs and the presence of the phonon drag effect.

Functionally graded thermoelectric leg was conceived by taking the advantage of the thermoelectric materials having optimal *zT*s at different temperature ranges. The fundamental concept is to synthesize a leg to have an optimum *zT* all the way from the hot-end to the cold-end through locally selecting a particular material, microstructure, or composition. It was demonstrated 13.8% generator efficiency was achieved using piece-wised functional (segmented) leg^17^. Following the same principle, cascade module uses several functional thermocouple stages and separated electric circuit for each stage to improve its efficiency within a large working temperature range.

Practically, when all the electrical and thermal energy losses and material compatibility issues at all the interfaces are accounted for, the improvements in device’s efficiency achieved at current scale are clearly insufficient for the realization of an efficient and an affordable device, hardly to replace traditional power generation systems. Furthermore, current device design and optimization can only work around the efficiency at the level dictated by the legs’ materials’ *zT* which is yet below the anticipated level, $zT>3$ within a working temperature range. Thus, the improvement of the efficiency has to be significant and beyond that limited by the material’s *zT*. In the author’s earlier work^2^, a new thermocouple design approach was proposed in an attempt to achieve its output power and efficiency beyond those of the legs’ materials’. A preliminary thermocouple design that uses low doping legs and high doping semiconductors as external carrier injectors surrounding the legs was then introduced and its potential in enhancing the device efficiency was demonstrated through an example.

In the current work, the proposed design approach is further elaborated with a new concept and the design details. In stead of enhancing the thermocouple’s *ZT*, an electrostatic voltage based on the *p-n* junction theory^18–21^, in addition to Seebeck voltage, is created in the thermocouple using the metallurgical junctions formed between the *n*- and the *p*-type legs and between the leg and the carrier emitters that are embedded on the legs’ exterior surfaces. A usable range of this electrostatic voltage and its effect on the output power and the efficiency were studied for different combinations of legs’ and emitters’ doping densities. Among these doping densities, an optimal combination was found, which yields the maximum output power and efficiency.

**Figure 1:**
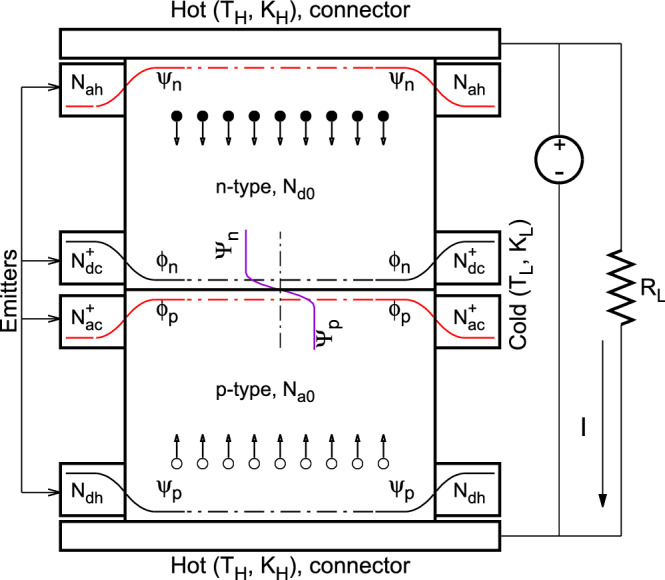
Diagram of the proposed new thermocouple design. The external carrier injectors or emitters surround the leg exterior surfaces. These emitters are labled by their doping densities and are enlarged for better illustration.

## Results

### New thermocouple design

#### Electrostatic voltage

The proposed thermocouple design is different from the conventional designs as shown in Fig. 1. It is composed of an *n*-type and a *p*-type semiconductor legs and four semiconductor emitters that act as carrier injectors of the two legs. The *n*- and the *p*-type legs, with their doping densities being denoted as $N_{d0}$ and $N_{a0}$, respectively, are connected directly at their cold ends so that a metallurgical junction (*p*-*n* junction) forms at the interface. Based on the *p*-*n* junction theory^18–21^, a built-in potential exists at such a junction and is determined by the induced electrostatic potentials ($\Psi _n$ and $\Psi _p$, respectively) in the neutral regions on the two sides of the junction as shown in the center of the figure. The four emitters are embedded on the exterior surface of these two legs, thus there exist two other *p-n* junctions formed at the interfaces between the legs and the emitters. One is at the hot-end of the *n*-type leg between the leg and a *p*-type emitter with a doping density $N_{ah}$. The induced positive electrostatic potential, $\psi _n$, in the neutral region on the leg side is shown in the figure. The other is at the hot-end of the *p*-type leg between the leg and an *n*-type emitter with a doping density $N_{dh}$. It induces a negative electrostatic potential, $\psi _p$, in the neutral region on the leg side as shown in the figure. The other two metallurgical junctions are formed between the same type semiconductor materials with different doping densities. One is at the cold-end of the *n*-type leg between the leg and a high-doping ($N^{+}_{dc}$) *n*-type emitter. Under the large gradient of carrier concentration between the two sides of the junction, electrons of the high-doping emitter diffuse through the junction and move into the low-doping leg. As a result, some uncompensated positive ions left behind in the emitter near the junction, thus a built-in potential is formed at the junction. The induced electrostatic potential in the neutral region on the leg side, $\phi _n$, is negative as shown in the figure. The other is at the cold-end of the *p*-type leg formed between the leg and a high-doping ($N^{+}_{ac}$) *p*-type emitter. Following the same mechanism as the *n*-type one, a positive electrostatic potential, $\phi _p$, is induced in the neutral region on the leg side. These induced electrostatic potentials form an electrostatic voltage between the two hot ends of the thermocouple.

Based on the *p-n* junction theory, these induced electrostatic potentials are determined by the following equations. The electrostatic potentials at the *p*-*n* junction between the legs are calculated by:

1a $$\begin{aligned}{\Psi _n} = - \frac{k_B\,T_c}{q_e}\ln \left[ \frac{N_{d0}}{n_i(T_c)}\right] \quad {\mathrm {in\; the\; neutral\; region \;of \;the}} \,\; {n}{\text{-}}{\mathrm{type\; leg}} \end{aligned}$$

1b $$\begin{aligned}{\Psi _p} = \frac{k_B\,T_c}{q_e}\ln \left[ \frac{N_{a0}}{n_i(T_c)}\right] \quad {\mathrm {in\; the\; neutral\; region\; of\; the}}\,\; { {p}}{\text{-}}{\mathrm{type\; leg}} \end{aligned}$$

where $k_B$ is the Boltzmann’s constant (in J/K), $q_e$ the electric charge (in Coulomb), $T_c$ the cold-end temperature (in Kelvin), $n_i$ the intrinsic carrier concentration (in cm$^{-3}$).

The electrostatic potentials at the two hot *p*-*n* junctions between the legs and their emitters are calculated by the same equations as Eq. (1) but at different temperatures:

2a $$\begin{aligned}{\psi _n} = - \frac{k_B\,T_h}{q_e}\ln \left[ \frac{N_{d0}}{n_i(T_h)}\right] \quad {\mathrm {in\; the\; neutral\; region \;of \;the}}\,\; { {n}}{\text{-}}{\mathrm{type\;leg}} \end{aligned}$$

2b $$\begin{aligned}{\psi _p} = \frac{k_B\,T_h}{q_e}\ln \left[ \frac{N_{a0}}{n_i(T_h)}\right] \quad {\mathrm {in\; the\; neutral\; region\; of \;the}} \,\;{ {p}}{\text{-}}{\mathrm{type\; leg}} \end{aligned}$$

where $T_h$ is the hot-end temperature.

For the electrostatic potentials at the two metallurgical junctions with the same *n*- or *p*-type semiconductor materials with different doping densities, it is assumed that the carrier density on leg side of the junction is the same as that of its emitter’s. These potentials are therefore calculated approximately by:

3a $$\begin{aligned}{\phi _n} = - \frac{k_B\,T_c}{q_e}\ln \left[ \frac{N^{+}_{dc}}{n_i(T_c)} \right] \quad {\mathrm {in\; the\; neutral\; region\; of\; the}} \,\;{ {n}}{\text{-}}{\mathrm{type\; leg}} \end{aligned}$$

3b $$\begin{aligned}{\phi _p} = \frac{k_B\,T_c}{q_e}\ln \left[ \frac{N^{+}_{ac}}{n_i(T_c)} \right] \quad {\mathrm {in\; the\; neutral\; region\; of\; the}}\,\; { {p}}{\text{-}}{\mathrm{type\; leg}}. \end{aligned}$$

The electrostatic potential difference (denoted as electrostatic voltage) between the two hot ends of the proposed thermocouple shown in Fig. 1 can then be determined by:

4 $$\begin{aligned} {V_i} = \left[ \psi _n - \left( \Psi _n - \phi _n \right) \right] - \left[ \psi _p - \left( \Psi _p - \phi _p \right) \right] \end{aligned}$$

This electrostatic voltage ($V_i$) is created by those metallurgical junctions formed between the *n*- and the *p*-type legs and between the legs and their emitters without any involvement of the thermoelectric phenomenon. Thus, it is an addition to the Seebeck voltage ($\alpha \,\Delta T$) and can boost the efficiency of the thermocouple without resorting to “tune” the thermoelectric transport properties. Assuming the leg doping density is independent of temperature, $\psi _n$ is actually smaller than $\Psi _n$ considering $n_i$ increases with temperature. Under such a scenario, $\phi _n$ plays an important role in contributing to the enhancement of output power and efficiency of the proposed thermocouple as will be discussed in the late sections.

#### Electric and heat currents

Within a thermoelectric leg which behaves like a battery, there exist both the induced electrostatic field ($\vec {E}$) and the non-electrostatic force ($\vec {{\mathscr {K}}}$) that drives the carriers to move through the leg from its hot-end to its cold-end. The direction of the non-electrostatic force is opposite to that applied by the induced electrostatic filed. The Ohm’s law for the current flux density (*j*) in the leg can thus be expressed as:

5 $$\begin{aligned} {\vec {j}} = \sigma _T({\vec {{\mathscr {K}}}} + {\vec {E}}) \end{aligned}$$

where $\sigma _T$ is the isothermal electrical conductivity.

The electromotive force (${\mathscr {E}}$) of a battery is defined as: *the work produced by a non-electrostatic force to move a unit positive charge from the negative terminal to the positive terminal through the battery*, i.e.,

6 $$\begin{aligned} {\mathscr {E}} = \int _{l^{-}}^{l^{+}} {\vec {{\mathscr {K}}}} \cdot d{\vec {l}} \end{aligned}$$

where $l^{-}$ and $l^{+}$ are the negative and the positive terminal positions, respectively, and $d\vec {l}$ is the incremental length vector of the circuit.

The terminal potential is defined to be equal to the work used to move a unit positive charge from the positive terminal to the negative terminal, i.e.,

7 $$\begin{aligned}U = U_{+} - U_{-} = \int _{l^{+}}^{l^{-}} {\vec {E}} \cdot d{\vec {l}}. \end{aligned}$$

Since in the battery ${\vec {E}}=-{\vec {{\mathscr {K}}}} + {\vec {j}}/{\sigma _T}$, Eq. (7) becomes:

8 $$\begin{aligned}U &= \int _{l^{+}}^{l^{-}} (- {\vec {{\mathscr {K}}}} + {\vec {j}}/{\sigma _T}) \cdot d{\vec {l}} = \int _{l^{-}}^{{{l^{+}}}} {\vec {{\mathscr {K}}}} \cdot d{\vec {l}} - \int _{l^{-}}^{l^{+}} (\rho j) dl \cos \theta \\&= \int _{l^{-}}^{l^{+}} {\vec {{\mathscr {K}}}} \cdot d{\vec {l}} - \int _{l^{-}}^{l^{+}} (j S) \frac{\rho dl}{S} \cos \theta = {\mathscr {E}} {-} I R_I \end{aligned}$$

where $I = j\,S$ is the electric current, *S* is the cross-sectional area of the circuit and $R_I = \int _{l^{-}}^{l^{+}} \rho dl/S$ the internal electrical resistance, where $\rho$ is the electrical resistivity.

In the proposed new thermocouple design, the electromotive force is the sum of the Seebeck voltage ($\alpha \,\Delta T$) and the electrostatic voltage induced by the metallurgical junctions, i.e.,

9 $$\begin{aligned}{{\mathscr {E}}} = \alpha \Delta T + V_i \end{aligned}$$

For the external circuit with load resistance $R_L$, $U = I R_L$. Introducing $U = I R_L$ into Eq. (8), the Ohm’s law is realized,

10 $$\begin{aligned} I = \frac{{\mathscr {E}}}{R_L + R_I} = \frac{{\mathscr {E}}}{R_T} \end{aligned}$$

where $$R_T = R_L + R_I$$.

The heat current equation thus becomes

11 $$\begin{aligned} I_q = \alpha T I + K_{np} \Delta T = \frac{\alpha T}{R_T} \left( \alpha \Delta T + V_i\right) + K_{np} \Delta T \end{aligned}$$

where $K_{np}$ is the leg thermal conductance and $\Delta T = T_h - T_c$.

#### Enhanced output power and efficiency

Assuming the external load resistance $R_L = m\,R_I$, the output power can then be expressed as:

12a $$\begin{aligned}P = IV = I^2\,R_L &= \frac{m}{R_I\,\left( 1 + m\right) ^2}\, \left( \alpha \Delta T + V_i\right) ^2 \\&= \frac{m}{R_I\,\left( 1 + m\right) ^2}\,\left[ \left( \alpha \,\Delta T\right) ^2 + 2\,\left( \alpha \,\Delta T\right) \, V_i + V_i^2\right] = P_{\alpha } + P_{\alpha \,V_i} + P_{V_i} \end{aligned}$$

where

12b $$\begin{aligned} P_{\alpha } = \frac{m}{R_I\,\left( 1 + m\right) ^2}\, \left( \alpha \,\Delta T\right) ^2 \end{aligned}$$

is the output power of the original thermocouple design without any external carrier injection.

12c $$\begin{aligned} P_{{\alpha }{V_i}} = \frac{2\,m}{R_I\,\left( 1 + m\right) ^2}\, \left( \alpha \,\Delta T\right) \,V_i \end{aligned}$$

is the output power generated by both Seebeck voltage and the electrostatic voltage induced by metallurgical junctions.

12d $$\begin{aligned} P_{V_i} = \frac{m}{R_I\,\left( 1 + m\right) ^2}\,V^2_i \end{aligned}$$

is the output power generated by the electrostatic voltage alone.

The efficiency of the thermocouple is calculated by:

13 $$\begin{aligned} \eta = \frac{P}{Q_h} = \frac{P_{\alpha }}{Q_h} + \frac{P_{{\alpha }{V_i}}}{Q_h} + \frac{P_{V_i}}{Q_h} = \eta _{\alpha } + \eta _{{\alpha }{V_i}} + \eta _{V_i} \end{aligned}$$

where $Q_h$ is the input heat rate. It need to emphases that among the three efficiency terms, only the $P_{\alpha }$ is totally generated through $Q_h$. The second term, $P_{{\alpha }{V_i}}$ is partially generated through $Q_h$. The third term, $P_{V_i}$ is not generated through $Q_h$. Thus, the efficiency of the proposed thermocouple could be greater than the following theoretical efficiency:

14 $$\begin{aligned} \eta _{theor} = \frac{T_h-T_c}{T_h}\, \frac{\sqrt{1+ZT}-1}{\sqrt{1+ZT}+\frac{T_c}{T_h}}. \end{aligned}$$

Depending on the value of $V_i$, four different cases can be expected for the output power of the new thermocouple design. (1) When $V_i < 0$, the built-in potential between the two legs ($\Psi _{np} = \Psi _n - \Psi _p$, positive) is larger than the compensating potential ($\phi _{np} = \phi _n - \phi _p$, negative). This will raise the thermoelectric potentials at the cold ends and thus lower the Seebeck voltage. The equation, Eq. (12), for the output power becomes:

15 $$\begin{aligned}P = \frac{m}{R_I\,\left( 1 + m\right) ^2}\,\left[ \left( \alpha \,\Delta T\right) ^2 - 2\,\left( \alpha \,\Delta T\right) \, |V_i| + V_i^2\right] = P_{\alpha } - P_{\alpha \,V_i} + P_{V_i}. \end{aligned}$$

Eventually, the output power will be less than that generated by the original thermocouple design if $-\alpha \Delta T \leqslant V_i < 0$. if $V_i < -\alpha \Delta T$, the output power will be produced by the $V_i$ against the thermoelectric driving force, which will not be discussed in the current work. For a proper combination of the doping densities of those emitters, such a condition can always be avoided. (2) When $V_i > 0$, $\Psi _{np} \leqslant |\phi _{np}|$. As a result, the potentials $\psi _{np} = \psi _{n} - \psi _p$ and $\phi _{np} - \Psi _{np}$ have the same polarities as the thermoelectric potentials. The output power is determined by Eq. (12), which will be larger than that of the original thermocouple design. This condition can be easily met without much effort in selecting a proper combination of the doping concentrations of those emitters. (3) When $V_i = \alpha \,\Delta T$, $\psi _{np} - (\phi _{np} + \Psi _{np}) = \alpha \Delta T$. Equation 12 becomes:

16 $$\begin{aligned}P = \frac{4\,m\,\left( \alpha \,\Delta T\right) ^2}{R_I\,\left( 1 + m\right) ^2} = 4\,P_{\alpha }. \end{aligned}$$

The output power will be four times that generated by the original design. In the late section, it will demonstrate that this condition can always be realized for different doping densities of the *n*- and the *p*-type legs through optimizing the doping densities of their emitters. Among these optimal doping combinations, there exists an optimum doping combination that can yield the maximum output power. (4) When $V_i > \alpha \,\Delta T$, the electric field force induced by the potentials of $\psi _n$ and $\phi _n + \Psi _n$ will outbalance the thermoelectric driving force ($\nabla T$) and pushs the carriers toward the direction opposite to the current flow driven by the thermoelectric driving forcei. With the reduction of the electric current, this condition will eventually lower the output power and should be avoid by a proper selection of the combination of doping concentrations of those emitters. In this case, Eq. (12) can not be directly used to determined the output power. Since the third condition, $V_i = \alpha \Delta T$, is the main interest of the current work and in practical application as well, the calculation of the output power for this condition will not be covered here.

The following equation can be used to evaluate the improving rate of output power:

17 $$\begin{aligned}\eta _P = \frac{P - P_{\alpha }}{P_{\alpha }} = \left[ \frac{2\,V_i}{\alpha \,\Delta T} + \left( \frac{V_i}{\alpha \,\Delta T}\right) ^2\,\right] \end{aligned}$$

It is clear that when $V_i = \alpha \,\Delta T$, the maximum improving rate of 3 can be obtained. The same improving rate can also be obtained for the thermocouple efficiency if the input heat rate is the same for both the original and the new thermocouple designs. This conclusion will be validated in the numerical simulation of the thermocouple in the late section.

### Thermocouple model description

#### Heat balance equation

Let us assume that all the parameters of the thermocouple shown in Fig. 1 are independent of temperature. The heat rates supplied to the hot side of the thermocouple ($Q_h$) and pumped out from its cold side ($Q_c$) are, respectively, given by:

18a $$\begin{aligned}Q_h = K_H \left( T_H - T_h\right) \end{aligned}$$

18b $$\begin{aligned}Q_c = K_L \left( T_c - T_L\right) \end{aligned}$$

where $K_H$ is the thermal conductance of the components from the heat source to the hot-end of the leg and $K_L$ is the thermal conductance of the components from the cold-end of the leg to the heat sink, $T_H$ is the heat source temperature, $T_h$ is the temperature at the hot end of the leg, $T_c$ is the temperature at the cold-end of the leg, and $T_L$ is the heat sink temperature.

The heat flow rates enter the hot-end and exit from the cold-end of the thermocouple are given by^22,23^:

19a $$\begin{aligned}Q_h = \alpha T_h I - \frac{1}{2} R_I I^2 + K_{np} \Delta T \end{aligned}$$

19b $$\begin{aligned}Q_c = \alpha T_c I + \frac{1}{2} R_I I^2 + K_{np} \Delta T \end{aligned}$$

where $K_{np}$ is the leg thermal conductance. The net heat flow rate through the thermocouple is calculated by subtracting Eq. (19b) from Eq. (19a):

20 $$\begin{aligned}Q_h - Q_c = \alpha \Delta T I - R_I I^2 \end{aligned}$$

Introducing Eqs. (18a) and (18b) into Eq. (20), one obtains:

21 $$\begin{aligned}\alpha \Delta T I - R_I I^2 = KT_{HL} -K_H \Delta T - K_T T_c \end{aligned}$$

where $KT_{HL} = K_H T_H + K_L T_L$ and $K_T = K_L + K_H$. Introducing Eq. (18b) into (19b) and rearranging the terms, one obtains:

22 $$\begin{aligned}\frac{1}{2} R_I I^2 + K_{np} \Delta T + K_L T_L = \left( K_L - \alpha I\right) T_c \end{aligned}$$

Finally, the following two equations are obtained to solve for $\Delta T$ and $T_c$:

23a $$\begin{aligned}\alpha \Delta T I - R_I I^2 - KT_{HL} + K_H \Delta T + K_T T_c = 0 \end{aligned}$$

23b $$\begin{aligned}\frac{1}{2} R_I I^2 + K_{np} \Delta T + K_L T_L = \left( K_L - \alpha I\right) T_c \end{aligned}$$

Substituting $T_c$ obtained from Eq. (23b) into (23a) and rearranging the terms give the following equation:

24 $$\begin{aligned}&- \alpha ^2 \Delta T I^2 + \alpha I \Delta T \left( K_L - K_H\right) + + \alpha R_I I^3 + \alpha I KT_{HL} + R_I I^2 \left( \frac{1}{2} K_T - K_L\right) \\& + \left( K_H K_L + K_{np} K_T\right) \Delta T + K_L \left( K_T T_L - KT_{HL} \right) = 0 \end{aligned}$$

Denoting $n = K_H/K_L$ and $\chi = K_L/K_{np}$, Eq. (24) can be further reduced to:

25 $$\begin{aligned}- \alpha ^2 I^2 \Delta T + \alpha R_I I^3 + \alpha I KT_{HL} + \left( \alpha I \Delta T - \frac{1}{2} R_I I^2 \right) \left( 1 -n\right) K_L + \left[ n \chi ^2 + \left( 1 + n\right) \chi \right] K_{np}^2 \Delta T - K_L K_H DT = 0 \end{aligned}$$

Substituting the electric current given by Eq. (10) and combining common terms, a cubic equation in terms of $\Delta T$ is obtained:

26a $$\begin{aligned}a \left( \Delta T \right) ^3 + b \left( \Delta T \right) ^2 + c \left( \Delta T \right) + d = 0 \end{aligned}$$

where

26b $$\begin{aligned}a = - \frac{m \alpha ^4}{\left( 1 + m \right) ^2 R_I^2} \end{aligned}$$

26c $$\begin{aligned}b = \left[ \frac{\left( 2 m +1\right) \alpha ^2}{2 R_I\left( 1 + m\right) ^2}\left( 1 - n\right) K_L - \frac{\alpha ^3 V_i \left( 2 m - 1\right) }{R_T^2 \left( 1 + m\right) ^3}\right] \end{aligned}$$

26d $$\begin{aligned}c = \left\{ \frac{\alpha ^2 V_i^2 \left( 2 - m\right) }{R_T^2 \left( 1 + m\right) ^3} + \frac{m \alpha V_i}{R_I \left( 1 + m\right) ^2}\left( 1 - n\right) K_L + \frac{\alpha ^2 K_L}{R_I \left( 1 + m\right) }\left[ n DT + \left( 1 + n\right) T_L\right] + \left[ n \chi ^2 + \left( 1 + n\right) \chi \right] K_{np}^2 \right\} \end{aligned}$$

26e $$\begin{aligned} d = \left\{ \frac{\alpha V_i^3}{R_I^2 \left( 1 + m\right) ^3} - \frac{V_i^2 \left( 1 - n\right) K_L}{2 R_I \left( 1 + m\right) ^2} + \frac{\alpha V_i K_L}{R_I \left( 1 + m\right) }\left[ n DT + \left( 1 + n\right) T_L\right] - n \chi ^2 K_{np}^2 DT \right\} \end{aligned}$$

Dividing all its coefficients of Eq. (26a) by *a*, a standard cubic equation is obtained:

27a $$\begin{aligned}\left( \Delta T \right) ^3 + B \left( \Delta T \right) ^2 + C \left( \Delta T \right) + D = 0 \end{aligned}$$

where

27b $$\begin{aligned}B = \frac{b}{a} = \left( 1 + \frac{1}{m}\right) \left( m + \frac{1}{2}\right) \left( n -1\right) \chi _Z + \left( 2 - \frac{1}{m}\right) V_{\alpha } \end{aligned}$$

27c $$\begin{aligned}C = \frac{c}{a} &= - \left( \frac{2}{m} - 1\right) V^2_{\alpha } + \left( 1 + m\right) \left( n - 1\right) V_{\alpha } \chi _Z - \frac{\left( 1 + m\right) ^2}{m} \chi _Z \left[ n DT + \left( 1 + n\right) T_L \right] \\&\quad - \frac{\left( 1 + m\right) ^3}{m}\left[ n \chi _Z^2 + \left( 1 + n\right) \frac{\chi _Z}{Z} \right] \end{aligned}$$

27d $$\begin{aligned}D = \frac{d}{a} = - \frac{V^3_{\alpha }}{m} + \left( 1 + \frac{1}{m}\right) \left( 1 - n\right) \frac{V^2_{\alpha }}{2} \chi _Z - \frac{\left( 1 + m\right) ^2}{m} V_{\alpha } \chi \left[ n DT + \left( 1 + n\right) T_L \right] + \left( 1 + m\right) ^3 \frac{n}{m} \chi ^2_Z DT \end{aligned}$$

where

27e $$\begin{aligned} V_{\alpha } = \frac{V_i}{\alpha } \quad \quad \chi _Z = \frac{\chi }{Z} \end{aligned}$$

#### Solving the cubic equation for $\Delta T$

There exists a standard method for solving a cubic algebraic equation. Here, a brief description is given for finding $\Delta T$ from Eq. (27a). The discriminant of the cubic equation is calculated by:

28a $$\begin{aligned} {\mathrm {discr}}= R^2+Q^3 \end{aligned}$$

where

28b $$\begin{aligned}Q=\frac{3 C - B^2}{9} \end{aligned}$$

28c $$\begin{aligned}R = \frac{2 B^3 - 9 B C + 27 D}{54} \end{aligned}$$

If ${\mathrm {discr}}>0$, $\Delta T$ is given by:

29a $$\begin{aligned}\Delta T = S + Y - \frac{B}{3} \end{aligned}$$

where

29b $$S = \sqrt[3]{{ - R + \sqrt {R^{2} + Q^{3} } }},\quad {\mathrm{and}}\quad Y = \sqrt[3]{{ - R - \sqrt {R^{2} + Q^{3} } }}.$$

If ${\mathrm {discr}}<=0$, the smallest difference of $DT-r_i$ is used as $\Delta T$, where $DT=T_H-T_L$, $r_i$ is the roots of the cubic equation, and *i* = 1, 2, 3, i.e.,

30a $$\begin{aligned}\Delta T = \min (DT-r_1, DT-r_2,DT-r_3) \end{aligned}$$

where

30b $$\begin{aligned}&r_1 = S + Y - \frac{B}{3}, \quad r_2 = -\frac{S + Y}{2} - \frac{B}{3} + (S - Y) \frac{\sqrt{3}}{2} {\mathbf {j}}, \\&r_3 = -\frac{S + Y}{2} - \frac{B}{3} - (S - Y)\frac{\sqrt{3}}{2} {\mathbf {j}}. \end{aligned}$$

or

30c $$\begin{aligned}r_1 = r_o \cos (\theta /3),\quad r_2 = r_o \cos (\theta /3 + 2 \pi /3),\quad r_3 = r_o \cos (\theta /3 + 4 \pi /3); \end{aligned}$$

30d $$\begin{aligned}\cos (\theta ) = -R/\sqrt{-Q^3}, \quad r_o = 2 \sqrt{-Q}. \end{aligned}$$

Once $\Delta T$ is determined, $T_c$ can be calculated by Eq. (22). The output power and the efficiency of the proposed thermocouple is calculated by Eqs. (12a) and (13), respectively.

**Figure 2:**
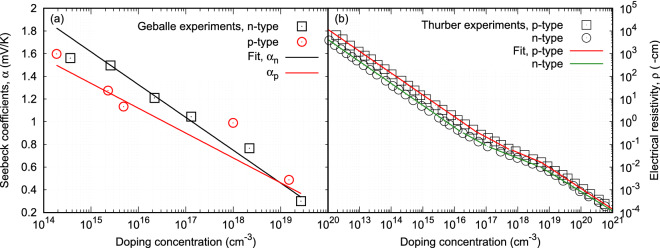
(**a**) Seebeck coefficients measured at different doping concentrations (data points)^24^. Lines are the coefficient fitting functions used in the thermocouple simulations. (**b**) Electrical resistivity measured at different doping concentrations and then fitted into the analytical functions (data points)^25^. Lines are the resistivity piece-wise fitting functions used in the thermocouple simulations.

## Discussion

A thermocouple with its leg length of 2 mm and its leg cross-sectional area of 0.267 cm$^2$ is used to study the output power and the efficiency of the proposed thermocouple design. Silicon semiconductor has mostly been used in making *p*-*n* junctions, thus is used for the study of the new thermocouple. For both *n*- and *p*-type Si semiconductors, their Seebeck coefficients measured at different doping concentration are given in Fig. 2a^24^. Their electrical resistivity as function of doping concentration are given in Fig. 2b^25^. Considering their thermal conductivity are relatively independent of their doping concentrations when the temperature is beyond 300 K, a constant value is assumed in the current study. For the *n*-type leg, its thermal conductivity is 100 W/m-K. For the *p*-type leg, its thermal conductivity is 200 W/m-K. The emitters in the new thermocouple design are only used for carrier injection, their doping concentrations are the only relevant parameters in the current study. Their doping concentration vary between $5\times 10^{14}$ cm$^{-3}$ and $5\times 10^{21}$ cm$^{-3}$. Since leg doping concentration has to be less than its emitters, it varies between 10$^{14}$ cm$^{-3}$ and $N_{dc}$ for the *n*-type leg and between 10$^{14}$ cm$^{-3}$ and $N_{ac}$ for the *p*-type leg. The parameters used in solving Eq. (26a) are: $T_H$ = 500 K, $T_L$ = 300 K, $\chi$ = 5, *n* = 1, and *m* = 1. For the cases considered in this work, the temperature difference between $T_h$ and $T_c$ obtained from Eq. (26a) is around 142 K.

**Figure 3:**
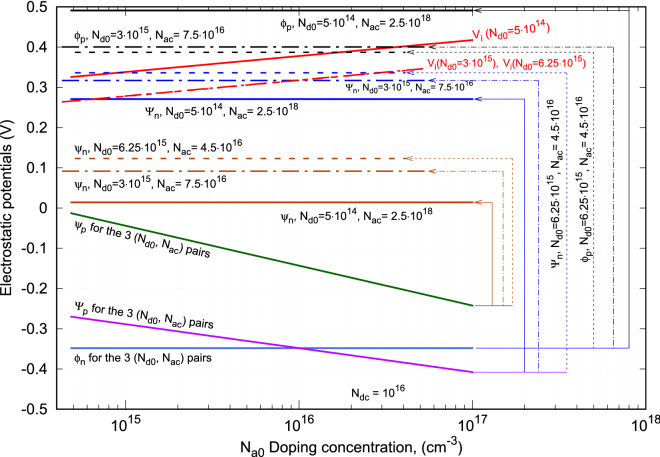
Variations of the electrostatic potentials and the electrostatic voltages with *p*-type leg doping concentration for a given doping concentration of the emitter at the cold-end of the *n*-type leg and three *n*-type leg doping concentrations and their corresponding doping concentrations of the emitter at the cold-end of the *p*-type leg.

### Variation of $V_i$ with doping concentration

Based on Eq. (4), the electrostatic voltage of the proposed thermocouple is a function of the electrostatic potentials at all its metallurgical junctions. For a given doping density of the emitter at the cold-end of the *n*-type leg, $N_{dc} = 10^{16}$ cm$^{-3}$ and three *n*-type leg doping densities, $N_{d0}= 5\times 10^{14}$ cm$^{-3}$, 3$\times$10$^{15}$ cm$^{-3}$, and $6.25\times 10^{15}$ cm$^{-3}$, $V_i$ was calculated and its variation and variations of all the electrostatic potentials at their metallurgical junctions with the *p*-type leg doping density ($N_{a0}$) are shown in Fig. 3. The label for each line identifies its electrostatic potential and the related doping densities used to obtain the line. Let us firstly consider the six solid electrostatic potential lines (for $N_{d0}= 5\times 10^{14}$ cm$^{-3}$) in this figure for their characteristics. These lines are the variations of the electrostatic potentials of (from the top to the bottom), $\phi _p$, $\Psi _n$, $\psi _n$, $\psi _p$, $\Psi _p$ and $\phi _n$, respectively with $N_{a0}$. Since $\phi _p$, $\Psi _n$, $\psi _n$, and $\phi _n$ are independent of $N_{a0}$, they show the straight lines along the axis of *p*-type leg doping density. Only $\psi _p$ and $\Psi _p$ vary with $N_{a0}$ and they decrease with the *p*-type leg doping density as shown. The vertical lines drawn between each pair of the potential lines of $\phi _p$ and $\phi _n$, $\Psi _n$ and $\Psi _p$, and $\psi _n$ and $\psi _p$ mark the potential differences between each pair of the electrostatic potentials. The potential difference between $\Psi _n$ and $\Psi _p$ is the built-in potential of the *p*-*n* junction between the two legs. As it can be observed graphically, this built-in potential increases with $N_{a0}$ and becomes quite large at higher *p*-type leg doping density. While this built-in potential, on one hand, can assist carrier transport through the thermocouple, it can also lower the magnitude of Seebeck voltage ($\alpha \,\Delta T$) on the other hand. Hence, it has to be compensated by the potential difference $\phi _p-\phi _n$ to a degree that the electrostatic voltage can play a large role in enhancing the power production of the thermocouple. The electrostatic voltage of the thermocouple is hence the algebraic summation of these three potential differences, i.e.:

31 $$\begin{aligned} V_i = \left( |\psi _n|+|\psi _p|\right) + \left( |\phi _n|+|\phi _p|\right) - \left( |\Psi _n|+|\Psi _p|\right) \end{aligned}$$

This electrostatic voltage of the thermocouple is shown in red solid line in the figure, which increases with *p*-type leg doping density and its slope is opposite to that of the Seebeck coefficient versus doping density that is decreases with leg doping density as shown in Fig. 2. Such a relationship between $V_i$ and the Seebeck coefficient is the key in enhancing the efficiency of a thermocouple.

As the *n*-type leg doping density $N_{d0}$ and the emitter doping density $N_{ac}$ vary, the electrostatic potentials related to these doping densities, $\phi _p$, $\Psi _n$, $\psi _n$, $\psi _p$, $\psi _p$ and $\phi _n$ vary as shown in the dashed lines for $N_{d0}=$ 3$\times$10$^{15}$ cm$^{-3}$ and $N_{ac}=$ 7.5$\times$10$^{16}$ cm$^{-3}$ and the dot-dashed lines for $N_{d0}=$ 6.25$\times$10$^{15}$ cm$^{-3}$and $N_{ac}=$ 4.5$\times$10$^{16}$ cm$^{-3}$ in the figure. Since $\phi _n$, $\Psi _p$ and $\psi _p$ are independent of both $N_{d0}$ and $N_{ac}$, they remain the same as $N_{d0}$ and $N_{ac}$ vary as shown. $\psi _n$ and $\Psi _n$ depend only on the *n*-type leg doping density. They both increase with $N_{d0}$ as shown in brown and blue lines. $\phi _p$ depends only on $N_{ac}$ and increases with the emitter doping density. Since $\Psi _p$ remains the same while $\Psi _n$ increases with $N_{d0}$ for the given $N_{dc}$, the potential difference between $\Psi _n$ and $\Psi _p$ increases with $N_{d0}$. Similarly, the potential difference between $\psi _n$ and $\psi _p$ also increases with $N_{d0}$. However, for the potential difference between $\phi _n$ and $\phi _p$, the results are slightly different. This is because a maximum output power occurs when $N_{d0}$ is large and $N_{ac}$ is small as can be noticed from the line labels (this is due the doping density rules to be discussed subsequently). As $N_{d0}$ increases, $N_{ac}$ has to decrease to attain an optimal output power. As a result, the potential difference between $\phi _n$ and $\phi _p$ decreases as $N_{d0}$ increases. Thus, $V_i$ decreases as $N_{d0}$ increases as shown in red dashed and dot-dashed lines in the figure. These two lines are overlapped.

The decrease of the potential difference between $\phi _n$ and $\phi _p$ with $N_{d0}$ is originated from the rules in determining a valid $V_i$ for enhancing the efficiency of the proposed thermocouple. These rules are designed as following. (1) $V_i$ has to be less than or at most equal to Seebeck voltage ($\alpha \,\Delta T$). This is because when $V_i > \alpha \,\Delta T$, carriers will move against thermoelectric driving force ($\Delta T$), thus lower the Seebeck voltage and the output power of the thermocouple. (2) The *n*-type leg’s doping density has to be less than that of its emitter, i.e., $N_{d0} < N_{dc}$ to maintain correct polarity of the electrostatic potentials of the metallurgical junction. For the same reason, *p*-type leg’s doping density has also to be less than that its emitter, i.e., $N_{a0} < N_{ac}$. (3) The minimal leg’s doping density should be greater or equal to the doping density where Seebeck coefficient attains maximum value.

Following these rules, the electrostatic voltage $V_i$ determined for different combinations of the doping densities, two legs and two emitters, will be valid and can make contribution in enhancing the output power and the efficiency of the proposed thermocouple.

**Figure 4:**
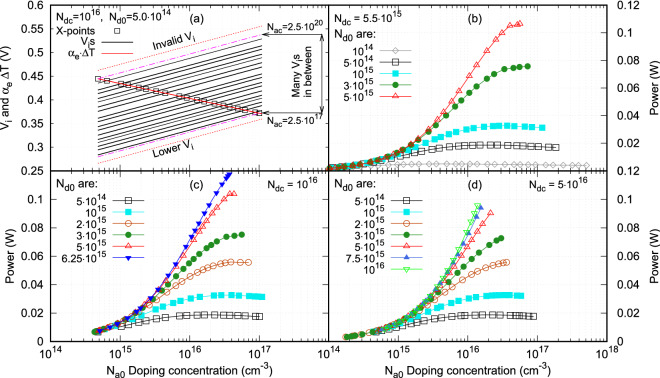
(**a**) Variations of Seebeck voltage and the electrostatic voltage with *p*-type leg doping density, $N_{a0}$. (**b**) Output power versus $N_{a0}$ for the emitter doping density, $N_{dc}=$ 5.5$\times$10$^{15}$. (**c**) Output power versus $N_{a0}$ for $N_{dc}=$ 10$^{16}$. (**d**) Output power versus $N_{a0}$ for $N_{ac}=$ 5$\times$10$^{16}$.

### Output power and efficiency

The output power, the efficiency, and the power improving rate of the proposed thermocouple were calculated via Eqs. (12), (13) and (17) for different legs’ and emitters’ doping densities. The results for output power are shown in Fig. 4. For a given $N_{dc}$ and a given $N_{d0}$, there exist numerous electrostatic voltages along the Seebeck voltage versus doping density curve, each corresponds to a specific $N_{ac}$, within a finite doping range of the *p*-type leg, $N_{a0}$, as shown in Fig. 4a. These $V_i$ lines parallel with each other and can thus be represented by a single linear function of log(x) and differ with one another only by a constant. Since these $V_i$s have a positive slope in the log($N_{a0}$)-V$_i$ coordinate system, they intercept with the curve of Seebeck voltage, i.e., $\alpha \,\Delta T$ which essentially has a negative slope. The Seebeck voltage curve separates the $V_i$ lines into upper and lower regions in the $V_i$’s slope direction. The $V_i$s located in the upper region are greater than $\alpha \,\Delta T$, thus are invalid based on the rules discussed in the previous section, while the $V_i$s in the lower region are less than $\alpha \,\Delta T$, thus are valid and can make contribution in enhancing the power production and efficiency of the thermocouple. Clearly, the $V_i$s on the Seebeck voltage curve have the equal values as $\alpha \,\Delta T$ at the interception points. At these interception points, the output power and efficiency reach their maximum values. Based on Eq. (12a), the output power (or efficiency) at the interception point is four times that of the conventional thermocouple design for the given legs’ doping densities. In Fig. 4a, there also exist two other regions in the direction perpendicular to the $V_i$’s slope, in which $V_i$ lines cannot find the interception with the Seebeck voltage curve based on the rules. These regions are either with invalid $V_i$s or with lower $V_i$s and are of no interest in the current study.

The output power at those $V_i = \alpha \,\Delta T$ points were calculated typically for $N_{dc}=$ 5.5$\times$10$^{15}$ cm$^{-3}$, 10$^{16}$ cm$^{-3}$, and 5$\times$10$^{16}$ cm$^{-3}$ and the results are shown, respectively, in Fig. 4b–d. There is a common characteristics among these three plots, that is, for a low doping *n*-type leg ($N_{d0}$), a peak output power exists along the power-$N_{a0}$ curve (the output power increases with $N_{a0}$ to a maximum value and then decreases with further increase of $N_{a0}$). This characteristics can be clearly observed particularly in Fig. 4b with low emitter doping density $N_{dc}=$ 5.5$\times$10$^{15}$ cm$^{-3}$. For all the *n*-type leg doping densities showed in this figure, a peak output power can be reached in all the power-$N_{a0}$ curves. For $N_{dc}=$ 10$^{16}$ cm$^{-3}$, five out of the six power-$N_{a0}$ curves have the peak output power. For $N_{dc}=$ 5$\times$10$^{16}$ cm$^{-3}$, three out of the seven power-$N_{a0}$ curves have the peak output power. The rest of the power-$N_{a0}$ curves do not have a peak point (or the peak point is at the end of the curve). By examining these three plots, Fig. 4b–d, it can be recognized that the largest output power occurs in Fig. 4c with $N_{dc}=$ 10$^{16}$ cm$^{-3}$, $N_{d0}=$ 6.25$\times$10$^{15}$ cm$^{-3}$, $N_{ac}=$ 4.125$\times$10$^{16}$ cm$^{-3}$, and $N_{a0}=$ 3.994$\times$10$^{16}$ cm$^{-3}$. These output power extrema depend on the relationship between the thermoelectric transport properties (Seebeck coefficient, electrical resistivity, and thermal conductivity) and the leg doping density. Thus, in this proposed thermocouple design, the output power and the efficiency not only depend on thermelectric transport properties but also depend on the electrostatic voltage ($V_i$). The electrostatic voltage should be a semiconductor device phenomenon and is independent of the thermoelectric phenomenon. Theoretically, it should not affect the thermoelectric transport properties. Under such a scenario, thermoelectric transport properties and the electrostatic voltage can be optimized individually to achieve a high-efficient thermoelectric generator.

From these results, it can be concluded that for a thermocouple with the given thermoelectric transport properties, an optimal combination of the emitter’s doping concentrations can always be found so that its output power and efficiency can be four times that of its conventional design.

**Figure 5:**
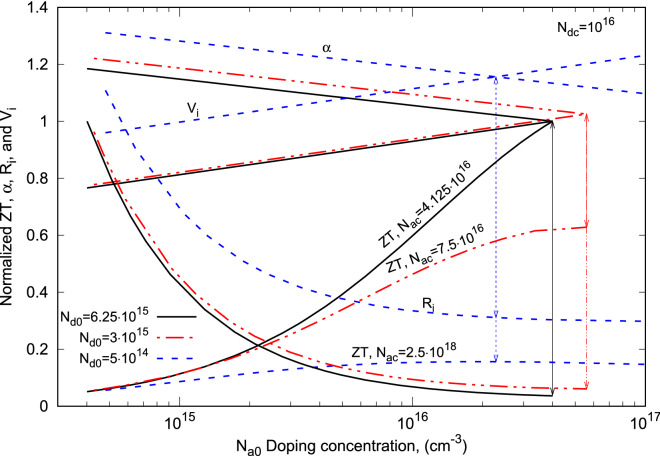
Thermoelectric transport properties, $\alpha$, resistance, ZT, and $V_i$ vary with the *p*-type leg doping density for three typical *n*-type leg doping densities.

An other common characteristics among these three plots, Fig. 4b–d, is that as $N_{dc}$ increases, the power-$N_{a0}$ doping density curves form two trends depending on the magnitude of $N_{d0}$. One is that for low *n*-type leg doping, the output power increases gradually with $N_{a0}$ from lower value to a maximum power and then decreases with further increase of $N_{a0}$, thus has a peak point (or P-$N_{a0}$ is a concave curve). The other is that for large $N_{d0}$, the output power increases monotonically with $N_{a0}$ without level off and attains a maximum P at the end of the curve (or P-$N_{a0}$ is a convex curve). This characteristics can be explained by examining the relations of the Seebeck coefficient, the electrical resistivity, the figure of merit, and the electrostatic voltage $V_i$ versus the *p*-type leg doping density as shown in Fig. 5. In Fig. 5, all the values, except $R_i$, are normalized with respect to the values corresponding maximum one of $N_{d0}=$ 6.25$\times$10$^{15}$ cm$^{-3}$. This plot shows the results for the emitter doping density $N_{dc}=$ 10$^{16}$ and three typical $N_{d0}$s that show different trends in the output power-$N_{a0}$ curves. Let’s firstly examine the results for the thermocouple group with $N_{d0}=$ 5$\times$10$^{14}$ cm$^{-3}$, showed in blue dashed lines in the figure. This thermocouple group has the largest Seebeck coefficient, electrical resistivity, and electrostatic voltage among those of the three thermocouples groups as shown. Its figure of merit (ZT) is the smallest one among the those of the three thermocouple groups. It increases gradually with $N_{a0}$ from lower values to a maximum ZT at $N_{a0}=$ 2.284$\times$10$^{16}$ and then decreases with further increase of $N_{a0}$. Its $V_i$ line intercepts with its Seebeck coefficient curve right at its maximum ZT point, which implies that the magnitude of the maximum output power of the thermocouple, the curve with square legends shown in Fig. 4c, is limited by the maximum figure of merit of the thermocouple. The thermocouple group with $N_{d0}=$ 3$\times$10$^{15}$ cm$^{-3}$ is represented by the red double-dot dashed lines. Its Seebeck coefficient, electrical resistivity, and electrostatic voltage are at the middle among those of the three thermocouple groups. Its ZT gradually increases with $N_{a0}$ and then level off and attains a maximum value right at the peak point of the curve (the peak point is also the last point in this case). Its $V_i$ line intercepts its Seebeck coefficient curve also right at the maximum ZT point, which means that its maximum output power is also limited by its maximum figure of merit. Since its ZT is much higher than that of the thermocouple group with $N_{d0}=$ 5$\times$10$^{14}$ cm$^{-3}$, thus a larger output power is obtained. The third thermocouple group with $N_{d0}=$ 6.25$\times$10$^{15}$ cm$^{-3}$ is represented by the black solid lines in the figure. Its Seebeck coefficient, electrical resistivity, and electrostatic voltage are the lowest among those of the three thermocouple groups, but its ZT is the highest. Its ZT increases monotonically with $N_{a0}$ up to the point where the maximum output power is reached. Its ZT does not reach its maximum value and can still increases with further increase of $N_{a0}$ but is cut off by the rule that $N_{a0}$ has to be smaller than $N_{ac}$. Its $V_i$ line intercepts its Seebeck coefficient curve right at the ZT cut off point. For this thermocouple group, the maximum output power is limited by the *p*-type leg doping concentration. Since the ZT value of this thermocouple at cut off point is much higher than those of the other two thermocouple groups, the highest output power is generated by this thermocouple.

As it can be observed in Fig. 5, the *p*-type leg doping density ($N_{a0}$) at which the maximum ZT is attained increases with the *n*-type leg doping density ($N_{d0}$) as marked by the vertical lines in the figure until it reaches a maximum value at which a maximum ZT can be attained with concave P-$N_{a0}$ curve without cutting off by $N_{a0}$. Afterwords, the *p*-type leg doping density starts to decrease with the increase of the *n*-type leg doping density. This characteristics is showed by the blue, red, and black vertical lines in Fig. 5. This characteristics can also be observed in Fig. 4b–d.

The output power showed in Figs. 4b–d actually varies with both $N_{a0}$ and $N_{ac}$. Thus, if continuous $N_{d0}$, $N_{a0}$ and $N_{ac}$ are considered for a fixed $N_{dc}$, the trends of the output power can be described mathematically. For a fixed $N_{dc}$, $N_{d0}$, $N_{a0}$, $N_{ac}$, and *ZT* form a 3D *ZT*-region with a mountain-like shape. $N_{a0}$ and $N_{ac}$ form the plane and *ZT* is the elevation. Each vertical cut section along a path of $N_{ac}$ and $N_{a0}$, ${\mathscr {L}}(N_{ac}, N_{a0})$, represents the variation of $ZT(N_{a0}, N_{ac})$ for a constant $N_{d0}$. Since for each $N_{d0}$, there exist a lower and an upper bound $N_{ac}$s and their corresponding $N_{a0}$s, as $N_{d0}$ varies in a given range, the bounds of $N_{ac}$ and $N_{a0}$ form a plane domain in which $V_i$, *ZT* and *P* are valid. This $N_{ac}$-$N_{a0}$ plane domain will cut the 3D *ZT*-region vertically into two sub-regions: valid and invalid. Within the valid *ZT*-region, the top *ZT* portion is cut off and certain local peaks on the hill remain. Thus, for large $N_{d0}$, the *ZT*-$N_{a0}$ curve cannot reach its peak *ZT* while for lower $N_{d0}$, a partial full *ZT*-$N_{a0}$ curve can still be obtainable. The output power can only be obtained within this valid *ZT*-region. As a result, the optimal doping density will be shifted from the original peak *ZT* to a position in the valid *ZT*-region where the maximum output power is achieved. Since within the valid *ZT*-region, $V_i$ can be added to the $\alpha \,\Delta T$, the maximum output power is higher.

Using the solution of $\Delta T$, Eqs. (29a) or (30), in the valid *ZT*-region, the maximum output power, Eq. (16), can be expressed as:

32 $$\begin{aligned}P = \frac{4\,m\,\left[ \alpha \,\left( S + Y - {B}/{3}\right) \right] ^2}{R_I\,\left( 1 + m\right) ^2}. \end{aligned}$$

where *S* and *Y* are defined by Eq. (29b). The optimal output power can be found by solving the following equation:

33 $$\begin{aligned} \frac{dP}{d(N_{a0})} = 0, \quad \quad N_{a0}^L \leqslant N_{a0} \leqslant N_{a0}^U; \quad ({\mathrm {along\; a\; path, }} {\mathscr {L}}(N_{a0}, N_{ac}), {\mathrm { with\; a\; constant }} \;N_{d0}\; {\mathrm { for\; a\; fixed }}\; N_{dc}) \end{aligned}$$

where $N_{a0}^L$ and $N_{a0}^{U}$ are the lower and upper bounds of $N_{a0}$.

The concave P-$N_{a0}$ curve can be expressed as:

34 $$\begin{aligned} \frac{d^2P}{d(N_{a0})^2}\left| _{N_{a0} = N_{a0}^{0}}\right. = 0, \quad \frac{d^2P}{d(N_{a0})^2}\left| _{N_{a0}< N_{a0}^{0}}\right.> 0, \quad {\mathrm {and}} \quad \frac{d^2P}{d(N_{a0})^2}\left| _{N_{a0} > N_{a0}^{0}}\right. < 0 \quad \quad {\mathrm {in}} \quad N_{a0}^L \leqslant N_{a0} \leqslant N_{a0}^U \end{aligned}$$

where $N_{a0}^0$ is a turning point.

The convex P-$N_{a0}$ curve can be expressed as:

35 $$\begin{aligned} \frac{d^2P}{d(N_{a0})^2} > 0, \quad \quad {\mathrm {in}} \quad N_{a0}^L \leqslant N_{a0} \leqslant N_{a0}^U. \end{aligned}$$

The border between these two trends is defined as:

36 $$\begin{aligned}\frac{dP}{d(N_{a0})}\left| _{N_{a0} = N_{a0}^{0}}\right. = 0 \quad \quad {\mathrm {and}} \quad \frac{d^2P}{d(N_{a0})^2}\left| _{N_{a0} = N_{a0}^{0}} = 0 \right. \quad {\mathrm {in}} \quad N_{a0}^L \leqslant N_{a0} \leqslant N_{a0}^U. \end{aligned}$$

## Conclusion

An electrostatic voltage can be formed through the electrostatic potentials at the metallurgical junctions created by the *n* and the *p*-type legs and their semiconductor emitters. This electrostatic voltage is an addition to the Seebeck voltage, thus can increase the output power and the efficiency of the proposed thermocouple up to four times those of the conventional thermocouple with the same leg doping densities.

For the given *n*- and *p*-type leg doping densities of a thermocouple, an optimal combination of the emitter doping densities can always be found such that the output power and the efficiency of the thermocouple with those emitters can be increased up to four times those of the conventional thermocouple without the emitters.

Since the heat rate supplied to the thermocouple is used by the thermoelectric driving force to move the carriers through the thermocouple and is not used by the electrostatic voltage to generate the output power, the efficiency of the proposed thermocouple can be greater than the theoretical efficiency derived from the laws of thermodynamics for thermoelectric generator.

## Methods

Seebeck coefficients obtained from experiments^24^ for both the *n*- and the *p*-type Silicon semiconductor materials were fitted into analytical functions for the use in the proposed thermocouple simulation. For the *n*-type Si, the fitting function is given by:

37 $$\begin{aligned} \alpha _n = 5.95208\times 10^3 - 2.89061\times 10^2\times \log (x) \end{aligned}$$

where *x* is the doping density (in cm$^{-3}$) of the material. Similarly, the fitting function for the *p*-type Si is obtained as:

38 $$\begin{aligned} \alpha _p = 4.6261\times 10^3 - 2.19187\times 10^2\times \log (x) \end{aligned}$$

These fitting functions were used in the simulation to calculate the effective Seebeck coefficient of the thermocouple by $\alpha _e = |\alpha _n| + \alpha _p$ for a given *n*-type and a given *p*-type leg doping densities.

The electrical resistivity measured from experiments^25^ for both the *n*- and the *p*-type Silicon semiconductor materials were also fitted into analytical functions piece-wisely using a linear interpolation of log(x) and log(y) functions, where *x* is the doping density of the material and *y* is the electrical resistivity. Using the piece-wisely fitting functions, the electrical resistivity for both the *n*- and the *p*-type Si were calculated in the simulation. The effective electrical resistivity of the thermocouple is then calculated by $\rho _e = \rho _n + \rho _p$.

Considering the thermal conductivity of both the *n*- and the *p*-type Silicon materials are nearly independent of the materials’ doping densities beyond 300 K, constants were used both the *n*- and the *p*-type Silicons in the simulation. For the *n*-type Si, $\kappa _n = 200$ (W/m-K) while for the *p*-type Si, $\kappa _p = 100$ (W/m-K). The effective thermal conductivity of the thermocouple is then given by $\kappa _e = \kappa _n + \kappa _p = 300$ (W/m-K).

For a given *n*-type leg doping density, $N_{d0}$ and a given doping density of its emitter, $N_{dc}$, using these thermoelectric transport properties of the thermocouple and the electrostatic voltage calculated from Eq. (4), the cubic equation (27) were solved for $\Delta T$ for a given range of doping desnity of the *p*-type leg, $N_{a0}$, and a given range of doping density of its emitter, $N_{ac}$.

From these calculations, variation of the effective Seebeck voltage with the *p*-type leg doping density can be determined. At the same time, variation of a group of electrostatic voltages for a range of emitter doping density, $N_{ac}$, with the *p*-type leg doping density were also obtained in these calculations. Both the Seebeck voltage and the group of electrostatic voltage were fitted into analytical functions. From these fitting functions, the interception points between these two voltages can be determined for each $N_{ac}$. A typical result is shown in Fig. 4a. Using these exact positions of the interception points of $\alpha \,\Delta T$-$V_i$ for each $N_{ac}$ in the specified range, an other simulation was conducted to determine the output power and the efficiency of the thermocouples at the interception points for the given $N_{d0}$ and the $N_{dc}$. The combination of these doping densities, $N_{d0}$, $N_{dc}$, $N_{a0}$, and $N_{ac}$ at each interception point yields the maximum output power and efficiency of the thermocouple. The largest one among these maximum output power gives an optimal combination of the doping densities for the given $N_{d0}$ and $N_{dc}$. These output power-$N_{a0}$ curves are shown in Fig. 4b–d for different $N_{d0}$ and $N_{dc}$s.

The largest output power among these curves yields the optimum combination of the doping densities, which gives the final thermocouple design.

## Data Availability

The datasets used and/or analysed during the current study are available from the corresponding author on reasonable request.
